# Snake Venom in Relation to Hemalysis, Bacteriolysis and Toxicity

**Published:** 1902-10

**Authors:** 


### Snake Venom in Relation to Hemalysis, Bacteriolysis and Toxicity.

Flexner and Noguchi, in the *University of Pennsylvania Medical Bulletin,* have an interesting article containing a *résumé* of experimental work done with dried venoms of the rattle snake, water moccasin, cobra and the copperhead. A final publication will be made later giving all the experiments and other work in detail; however, the above article gives the results of the investigation to date, together with much data concerning this very important research. C. R. M.

